# Chiropractic wellness on the web: the content and quality of information related to wellness and primary prevention on the Internet

**DOI:** 10.1186/2045-709X-19-4

**Published:** 2011-02-02

**Authors:** Marion Willard Evans, Stephen M Perle, Harrison Ndetan

**Affiliations:** 1 Texas Chiropractic College, Pasadena, TX, USA; 2 University of Bridgeport, Bridgeport, CT USA; 3 Parker Research Institute, Dallas, TX, USA

## Abstract

**Background:**

The Internet has become a common source of information for patients wishing to learn about health information. Previous studies found information related to back pain poor and often contradictory to current guidelines. Wellness has become a common topic in the field of chiropractic and accrediting agencies have standards on delivery of wellness-based content in college curricula as well as directives for clinical applications. The purpose of this study was to evaluate the quality of the information on the Internet using the terms "chiropractic wellness," or "wellness chiropractic".

**Methods:**

Five commonly used search engines were selected and the first 10 sites found using the strategy above were evaluated by two raters. Demographic assessments of the sites were made along with whether they were Health on the Net Foundation (HON) certified, contained standard wellness content, mentioned any Healthy People Focus Areas, and other chiropractic topics. Kappa statistics compared inter-rater agreement.

**Results:**

Potential patients appeared to be the audience 87% of the time and a private doctor of chiropractic appeared to be the typical site owner. The sites usually promoted the provider. No sites displayed HON certification logo nor did any appear to meet the HON certification criteria. Twenty-six sites (55%) promoted regular physical activity in some manner and 18 (38%) had information on health risks of tobacco. Four (9%) had mental health or stress-reduction content but none had information supportive of vaccination. Some had information contradictory to common public health measures.

**Conclusions:**

Patients searching the Internet for chiropractic wellness information will often find useless information that will not help them maintain health or become well. Most simply market the chiropractic practice or allow for a patients to provide personal information in exchange for more 'wellness' information. More research should be done on how providers determine site content, pay any attention to the details on their sites, or agree with content as some appear to be prefabricated sites. Website content could be enhanced by sharing of information from reputable sources like US Centers for Disease Control, the National Institutes of Health and other authoritative sources. HON certification should also be sought.

## Background

The Internet has become a common source of information for patients wishing to learn about health and medical information [1-4]. Internet sites can now be set up by virtually anyone and may contain information simply chosen at will by the site developers. Butler and Foster found information related to back pain on the Internet to be of poor quality with information often contradictory to current guidelines for management of back pain [5]. Studies on home management of childhood fever and depression on the Internet were also rated poorly in separate studies [6,7].

Specific evaluations of Internet-based information have been performed for back and spinal pain, scoliosis and spine-related education [8-10]. Li and colleagues searched the Internet for information on back pain using a search strategy of what a typical patient might utilize and found a lack of quality control, problems with credibility of information on the sites, and a minority of sites containing evidence-based information related to back pain [8]. Mathur and associates investigated the quality of information related to management of scoliosis on Internet sites and stated that the information was typically of limited quality and of poor information value [9]. They suggested that physicians assume more responsibility for the content of information on their sites. In a review of readability of sites related to spine-related educational information on websites reading levels were typically way above recommended levels averaging over the 10^th ^grade reading level [10].

Wellness has become a common topic in the field of chiropractic and the accrediting agency for chiropractic degree programs has standards on delivery of wellness-based content in college curricula as well as directives for practical application at the clinical level [11]. In addition, the United States (US) has established goals for the nation through the ***Healthy People ***(HP) initiatives that should guide content provided by all health care providers and community health programs related to wellness and primary prevention [12]. The Association of Chiropractic Colleges and the American Chiropractic Association, the largest trade group in the US for the chiropractic profession have mission statements supportive of these efforts [13,14]. The Chiropractic and Osteopathic College of Australasia has within its objectives to; "promote the integration of chiropractors and osteopaths into the broader community health team" and; to, "participate in activities related to public health" [15]. Wellness content then should be content that is congruent with the recommendations of such documents mentioned above, not in opposition to accepted public or community health measures, and not simply advertisement for services in a provider's office. The purpose of this study was to specifically evaluate the quality of the information patients searching the Internet with the terms "chiropractic wellness," or "wellness chiropractic" would find based on whether the information is in line with current, accepted health promoting measures related to general health and wellness described above. Further, sites were evaluated to see if they followed the criteria called for by Health on the Net Foundation (HON) http://www.hon.ch/ which provides guidelines for responsible health and medical information sites on the Internet. Sites were also assessed as to whether they contained typical chiropractic terms such as subluxation, innate intelligence, or spinal care and whether the presence of these terms would predict more or less health promotion and wellness advice available on the site.

## Methods

The search terms selected were "chiropractic wellness," and "wellness chiropractic." These terms are commonly used in signage on chiropractic offices in marketing materials so the rationale or aim of the investigators was to see what content this would net on the World Wide Web and evaluate that content. Five search engines commonly used by laypersons were selected. These were Google.com, Yahoo.com, Microsoft Bing.com, Ask.com, and AOL.com. These were selected because of their rankings as the most used websites from http://www.comscore.com. The first 10 sites found anywhere on the web by using the strategy above on each search engine were selected for evaluation.

Demographic assessments of the sites were made such as determining the audience they were aimed at, the source of the website, the purpose, whether they were Health on the Net Foundation (HON) certified http://www.hon.ch/, whether they contained standard wellness content, mentioned any HP Focus Areas, HP Ten Leading Health Indicators, and typical chiropractic topics. The typical chiropractic topics determined if the site mentioned subluxation, prevention of spinal pain, innate or presented anti-vaccination, anti-drug or anti-medical viewpoints. The site was judged using the 8 principles defined by HON:

1--Information must be authoritative giving qualifications of authors

2--Purpose of the website should be apparent

3--Confidentiality that respects the privacy site users

4--Information must be documented: citing sources and dates of medical information

5--Justification of claims

6--Website contact details should be apparent

7--Disclosure of funding sources

8--Advertising policy stated that clearly distinguishes advertising from editorial content

Data about websites were placed in an Excel spreadsheet with assessment of each of the criteria described. Assessment was made separately by two researchers (Rater1 and Rater2). Data was reviewed and converted into SPSS version 16 (SPSS Inc, Chicago, IL) for analysis. Primarily, frequencies were generated on the web content of each site as reviewed by Rater1 and Rater2. Kappa statistics was used to assess agreement beyond chance between the two reviewers. Kappa value is reported as a percent and commonly interpreted as follows: <20% negligible improvement over chance agreement alone, 21-40% minimal improvement, 41-60% fair, 61-80% good improvement, 81-92% very good, and 93-100% excellent [16]. The main chiropractic topic assessed was whether or not a webpage specifically mentioned subluxation, innate and/or spinal pain. Odds ratios (OR) and 95% confidence intervals (CI) were computed from binary cross-tabulations that assessed whether or not sites that mentioned each of those chiropractic topics (such as 'subluxation', 'innate', or management of spinal conditions) were also likely to mention anti-vaccination, anti-drug, or anti-medical information. It should be stated that the term 'subluxation' is a chiropractic term suggestive of nerve interference occurring when spinal alignment or function is altered by such events as trauma and that innate refers to the body having an innate or inborn ability to heal itself if spinal nerve interference is not present. These terms are unique to chiropractic in a historical sense and are commonly used by some practitioners adhering to a traditional belief that spinal conditions are a source of other diseases; hence, the interest in the evaluators as to presence or non-presence of these terms and their potential for effect modification of any analysis.

## Results

### General purpose and content of the site

A total of 47 unique websites were obtained from the 5 search engines for testing. All of the sites appear to be sites in North America. Nineteen sites were captured under the term 'chiropractic wellness,' and 18 with the term 'wellness chiropractic' and 10 of these were found with both search phrases. The results reported below are mainly based on one reviewer (Rater2), however, details on both reviewers as well as percent agreement between them are present in the tables.

Potential patients appeared to be the audience the websites were aiming at 87% of the time and a private doctor of chiropractic appeared to be the site owner 87% of the time. The purpose of the site was typically to promote the provider (87% of sites). None of the web sites displayed HON certification logo nor did any appear to meet the HON certification criteria. One site appeared to offer authoritative information and 1 stated the purpose of the site. Thirteen had statements related to confidentiality of the site. Only 2 had referenced and dated content and 2 had justification of claims. Contact details were unavailable on >90% of sites. Demographics of websites and Rater1 and 2 responses with kappa statistics appear in table 1.

**Table 1:** General website demographic information and rater responses

Variable	Rater1 n(%)	Rater2 n(%)	Kappa(%)
***Audience***			
Patient	44(94)	41(87)	100
Chiropractor	3(6)	3(7)	
Gen public	0(0)	2(4)	
Missing	0(0)	1(2)	
***Owner***			
Chiropractor	43(92)	41(87)	100
Non-profit	2(4)	3(7)	
Commercial	2(4)	2(4)	
Missing	0(0)	1(2)	
***HON certified***			
Yes	0(0)	0(0)	100
No	47(100)	47(100)	
***Info is authoritative***			
Yes	0(0)	1(2)	100
No	47(100)	46(98)	
***Purpose defined***			
Product	3(6)	1(2)	98
Service	0(0)	2(4)	
Provider	44(94)	41(88)	
Profession	0(0)	2(4)	
Missing	0(0)	1(2)	
***Confidentiality***			
Yes	0(0)	13(28)	72
No	47(100)	34(72)	
***Info documented, referenced, dated***			
Yes	0(0)	2(4)	96
No	47(100)	45(96)	
***Justification of claims***			
Yes	0(0)	2(4)	96
No	47(100)	45(96)	
***Website contact details***			
Yes	0(0)	2(6)	94
No	47(100)	44(94)	
***Discloses funding***			
Yes	0(0)	0(0)	100
No	47(100)	47(100)	
***Advertising policy***			
Yes	0(0)	1(2)	98
No	47(100)	46(98)	

### General health-related content on the site

Twenty-six sites (53%) promoted regular physical activity in some manner and 18 (38%) had information on tobacco or health risks of smoking. Four (9%) had mental health or stress-reduction content but none had information supportive of vaccination. Other data on general health information is noted in table 2 along with both raters' assessments and kappa statistics.

**Table 2:** General health and wellness topics mentioned on websites

Variable	Rater1 n(%)	Rater2 n(%)	Kappa (%)
***Site promotes physical activity***			
Yes	17(36)	26(53)	83
No	30(64)	22(47)	
***Smoking cessation information***			
Yes	2(4)	18(38)	66
No	45(96)	29(62)	
***Mental health and stress reduction***			
Yes	9(19)	4(9)	89
No	38(81)	43(91)	
***Safety (helmet, seatbelt use*)**			
Yes	1(2)	0(0)	98
No	46(98)	47(100)	
***Healthy diet***			
Yes	15(32)	27(57)	74
No	32(68)	20(43)	
***Alcohol use in moderation***			
Yes	0(0)	0(0)	100
No	47(100)	47(100)	
***Safe sex practices***			
Yes	0(0)	0(0)	100
No	47(100)	47(100)	
***Substance abuse***			
Yes	0(0)	0(0)	100
No	47(100)	47(100)	
***Supportive of vaccines***			
Yes	0(0)	0(0)	100
No	47(100)	47(100)	

### Healthy People content areas

No website specifically mentioned the topic of HP 2010 but 25 (53%) had some content that fell loosely under one of the HP headings. For example, one site had some content related to cancer prevention and one had information on prevention of stroke or heart disease but none had information specific to prevention of kidney disease, disability, secondary conditions, or family planning. Content related to prevention falling under HP Focus areas was rare on all of the websites reviewed. Table 3 lists content-specific information related to HP Focus areas.

**Table 3:** Healthy People content information found on websites

Variable	Rater1 n(%)	Rater2 n(%)	Kappa (%)
***Site mentions HP initiatives***			
Yes	0(0)	0(0)	100
No	47(100)	47(100)	
***HP Focus Areas mentioned***			
Yes	25(53)	25(53)	100
No	22(47)	22(47)	
***Access to quality health care***			
Yes	0(0)	0(0)	100
No	47(100)	47(100)	
**Conditions *Arthritis, osteoporosis, chronic back pain***			
Yes	9(19)	0(0)	81
No	38(81)	47(100)	
***Cancer prevention***			
Yes	0(0)	1(2)	98
No	47(100)	46(98)	
***Kidney Disease***			
Yes	0(0)	0(0)	100
No	47(100)	47(100)	
***Diabetes***			
Yes	3(6)	0(0)	94
No	44(94)	47(100)	
***Disability, secondary cond.***			
Yes	0(0)	0(0)	100
No	47(100)	47(100)	
***Educ. and community-based programs***			
Yes	0(0)	0(0)	94
No	44(94)	47(100)	
***Vision and hearing problems***			
Yes	0(0)	0(0)	100
No	47(100)	47(100)	
**Select Leading Health Indicators**			
***Physical activity***			
Yes	17(36)	25(53)	83
No	30(64)	22(47)	
*** Overweight/obese***			
Yes	9(19)	21(45)	74
No	38(81)	26(55)	
***Tobacco use***			
Yes	2(4)	8(17)	87
No	45(96)	39(83)	
***Responsible sexual behavior***			
Yes	0(0)	0(0)	100
No	47(100)	47(100)	
***Mental health***			
Yes	1(2)	10(21)	81
No	46(98)	37(79)	
***Injury and violence***			
Yes	1(2)	1(2)	100
No	46(98)	46(98)	
***Immunization***			
Yes	0(0)	0(0)	100
No	47(100)	47(100)	

Among the HP Leading Health Indicators, 25 (53%) sites specifically mentioned a topic that would fall under one of them. Some mention of physical activity (PA) was noted on 26 (53%) of sites and 21 (45%) mentioned issues related to overweight and obesity. As stated, 18 (38%) had some information related to the health issues associated with tobacco use. Only 1 site contained any information on injury and violence prevention and no site had information on the benefits of immunization, responsible sexual behavior, vision and hearing problems, or substance abuse.

### Chiropractic topic information

Thirty-six (77% of sites) contained information on chiropractic 'subluxation' and 28 (60%) had information on 'innate.' Thirty-one (66%) sites had information related to prevention of spinal pain. Regarding information expressing negative aspects of medical interventions or vaccines, 16 (34%) of sites contained obvious anti-vaccination information, 16 (34%) had information that was anti-drug (prescription or medical use of drugs), and 14 (30%) had general anti-medical information related to the medical profession. Interestingly, no site had specific HP Focus Area information on the topic of arthritis, osteoporosis, and chronic back conditions. Complete responses from reviewers and kappa ratios of chiropractic information are listed in table 4.

**Table 4:** Chiropractic characteristics and demographic information from websites

Variable	Rater1 n(%)	Rater2 n(%)	Kappa (%)
***Site specifically mentions subluxation***			
Yes	35(75)	36(77)	99
No	12(25)	11(23)	
***Site specifically mentions innate***			
Yes	34(72)	28(60)	87
No	13(28)	19(40)	
***Mentions preventing spine pain***			
Yes	37(79)	31(66)	87
No	10(21)	16(34)	
***Anti-vaccine information in website***			
Yes	14(30)	16(34)	96
No	33(70)	31(66)	
***Anti-medical information on website***			
Yes	19(40)	14(30)	89
No	28(60)	33(70)	
***Anti-drug information on website***			
Yes	19(40)	16(34)	94
No	28(60)	31(66)	

Sites that mentioned 'subluxation' were more likely to have anti-vaccine information compared to sites that did not [OR = 5.7(95% CI,0.6-50.6)], were more likely to have anti-drug information [OR = 5.7(95%CI,0.6-50.6)], and to have anti-medical information [OR = 4.5(95%CI,0.5-40.3)]. However, these did not approach statistical significance due to small cell numbers. Sites that mentioned 'innate' were more likely to contain anti-vaccine information [OR = 7.5(95%CI,1.4-39.0)], were more likely to have anti-drug information [OR = 7.5(95%CI,1.4-39.0)], and more likely to have anti-medical information on them as well [OR = 2.0(95%CI,1.4-2.9)]. Sites that mentioned spinal pain were more likely to contain anti-vaccine information as well when compared to those that did not, [OR = 4.9(95%CI, 0.9-26.0)], more likely contained anti-drug information [OR = 12.1(95%CI,1.4-105.0)], and more likely to contain anti-medicine information, [OR = 9.4(95%CI,1.0-81.0)], though not all were statistically significant at the 95% confidence level.

## Discussion

Reviewer agreement levels were generally good to excellent. In cases where variation occurred it was often due to the depth of site review. Some information may have appeared in the opening page of a website but on others, information was on panels that required the reviewer click several tabs to reach it. When information was not visible on the website in a prominent manner it was harder to locate and probably accounts for some variation between reviewers. In addition, reviewers used their own judgment as to whether sites contained information consistent with HP initiatives. Perhaps a good example is diet and health. Some sites contained information on diet and health. However, many of those were to market a nutritional product or supplement and may not have indicated a level of dietary information related to HP initiatives. This may have accounted for variation among reviewers in general.

The Internet will get more and more use as a potential source of information on health and wellness. Smart-phone technology now allows a search from almost anywhere. The purpose of this study was to see what wellness-oriented content would appear on sites the public would likely come across on a routine search of chiropractic wellness. The findings presented here indicate a lack of sound wellness and health promotion information available on most of the sites analyzed.

Research into what consumers look for to determine the credibility of an Internet site may be a cause for more concern over content. One study found that people look more at the professional design of the site, whether they can get, "a quick lay-out of the site", the user-friendliness of the site, and speedy interface times rather than if the content is evidence-based or supported by the scientific literature [17]. This is unfortunate but understandable. After all, these are laypersons and not providers. This same study found that patient markers for quality included if it "sounded plausible," sounded "scientific," looked "trustworthy." These have little to do with actual viability of content.

The depth of information on the sites analyzed was poor and was rarely evidence-based. Many sites may have mentioned one should walk for example but there was no evidence of information related to PA Guidelines for Americans [18] and information related to how one could get PA into their daily lives. This study also demonstrates that some chiropractors (those with web sites that are highly ranked by the search engines used) apparently still support the notion that chiropractic care is a viable substitute for medical care. Anti-medical, anti-drug, and anti-vaccine rhetoric was often displayed prominently on the sites evaluated. This is concerning. Worth noting is that several sites had very near identical content apparently having purchased a pre-manufactured website from one of a small number of vendors. Many practitioners may be purchasing these and paying little attention to the information that is on the sites. Nevertheless, content was poor on most sites lacking legitimate evidence-based wellness information. This is ultimately the responsibility of the provider.

Patients need information on how to become healthy and stay healthy. Many of the health conditions listed in HP documents and that this project assessed are the common causes of premature morbidity and mortality in Americans and typically, in a minority of sites was there any information that would likely help a patient restore or gain healthy ground. The sites typically marketed the chiropractor and offered promotional materials for services offered in the clinic and not wellness measures common to preventive medicine or health promotion. Even specific information on prevention of reoccurrence of back pain was scant on most sites. The anti-public health messaging could actually be seen by some as anti-prevention, therefore anti-wellness. This was discouraging as well since it could be seen on approximately 1/3 of sites reviewed.

### Limitations of the study

There are some specific limitations of this study. First, this was a review of a minimum number of sites and they may not be representative of all chiropractic sites. Second, two reviewers who are researchers made the reviews and there is bias inherent to that process. However, agreement was consistent most of the time. Third, many content areas related to HP initiatives clearly would not be expected to be found on a site to promote an individual chiropractor as was the apparent intent of most of the websites reviewed. Therefore, content like safe sex practices, vision and hearing issues are not likely to be found here even if they might benefit patients who view the sites. Lastly, all of the sites netted by the search on these most popular search engines appear to be in North America and therefore, we cannot assume any of the results would generalize to Europe, Australia, or other parts of the world.

## Conclusions

Patients searching the Internet for chiropractic wellness information will often find a lot of poorly done, useless information that will not help them maintain health or become well. Most of the time it simply markets the chiropractic practice and lists services that they offer in the office or allows for a patient to provide personal information in exchange for more 'wellness' information. This equates to marketing and practice building. More research should be done on how providers determine site content, whether they pay any attention to the details presented on their sites, and whether they even agree with it. Website content could be enhanced by sharing of information from reputable sources like the US Centers for Disease Control and Prevention, the National Institutes of Health and other authoritative sources. HON certification should be sought as well. Information that is illogical or contradictory to current, accepted public health and wellness practices should be removed to better serve patients and potential patients who visit these sites. This could serve to improve the cultural authority of chiropractic as providers interested in wellness in the process. Regulatory boards should begin to evaluate the advertising content on these sites as some of it seems to violate board rules on ethics and advertising.

## List of abbreviations

AOL: America Online search engine; Cl: confidence interval; HON: Health on the Net Foundation; HP: Health People initiative of the United States; OR: odds-ratio; PA: physical activity; US: United States.

## Competing interests

The authors are all primarily researchers and academics and acknowledge no competing interests related to the research presented.

## Authors' contributions

SMP conceptualized the idea for the study, developed the spreadsheet for comparative analysis, was a reviewer of websites, and helped construct and review the manuscript. MWE assisted in development of methods of assessment, reviewed the websites, assisted in data analysis and interpretation, and primarily constructed the manuscript. HN performed the statistical analysis, helped create all tables. All authors reviewed and edited the manuscript and approved the final manuscript.
